# Absenteeism and Health Behavior Trends Associated With Acute Respiratory Illness Before and During the COVID-19 Pandemic in a Community Household Cohort, King County, Washington

**DOI:** 10.1016/j.focus.2024.100248

**Published:** 2024-06-06

**Authors:** Erin Chung, Yongzhe Wang, Eric J. Chow, Anne Emanuels, Jessica Heimonen, Constance E. Ogokeh, Melissa A. Rolfes, James P. Hughes, Timothy M. Uyeki, Lea M. Starita, Samara Hoag, Michael Boeckh, Janet A. Englund, Helen Y. Chu, Seattle Flu Study Investigators

**Affiliations:** 1 Department of Pediatrics, University of Washington, Seattle Children's Hospital, Seattle, Washington; 2 Division of Allergy and Infectious Diseases, Department of Medicine, University of Washington, Seattle, Washington; 3 Public Health - Seattle & King County, Seattle, Washington; 4 Department of Epidemiology, University of Washington, Seattle, Washington; 5 Influenza Division, National Center for Immunization and Respiratory Diseases, Centers for Disease Control and Prevention, Atlanta, Georgia; 6 Military and Health Research Foundation, Laurel, Maryland; 7 Vaccine and Infectious Disease Division, Fred Hutchinson Cancer Research Center, Seattle, Washington; 8 Department of Biostatistics, University of Washington, Seattle, Washington; 9 Brotman Baty Institute for Precision Medicine, Seattle, Washington; 10 Department of Genome Sciences, University of Washington, Seattle, Washington; 11 Student Health Services, Seattle Public Schools, Seattle, Washington; 12 Fred Hutchinson Cancer Research Center, Seattle, Washington; 13 University of Washington School of Medicine, Seattle, Washington

**Keywords:** COVID-19, illness behavior, non-pharmaceutical interventions, absenteeism, viral infections, households

## Abstract

**Introduction:**

Longitudinal data on how acute respiratory illness (ARI) affects behavior, namely school or work participation, and nonpharmaceutical intervention (NPI) usage before and during the COVID-19 pandemic is limited. The authors assessed how ARIs and specific symptoms affected school, work, and health-related behaviors over time.

**Methods:**

From November 2019 to June 2021, participating households with children in King County, Washington, were remotely monitored for ARI symptoms weekly. Following ARIs, participants reported illness-related effects on school, work, and NPI use. Using logistic regression with generalized estimating equations, the authors examined associations between symptoms and behaviors.

**Results:**

Of 1,861 participants, 581 (31%) from 293 households reported 884 ARIs and completed one-week follow-up surveys. Compared with the prepandemic period, during the period of the pandemic pre–COVID-19 vaccine, ARI-related school (56% vs 10%, *p*<0.001) absenteeism decreased and masking increased (3% vs 28%, *p*<0.001). After vaccine authorization in December 2020, more ARIs resulted in masking (3% vs 48%, *p*<0.001), avoiding contact with non-household members (26% vs 58%, *p*<0.001), and staying home (37% vs 69%, *p*<0.001) compared with the prepandemic period. Constitutional symptoms such as fever were associated with work disruptions (OR=1.91; 95% CI=1.06, 3.43), staying home (OR=1.55; 95% CI=1.06, 2.27), and decreased contact with non-household members (OR=1.58; 95% CI=1.05, 2.36).

**Conclusions:**

This remote household study permitted uninterrupted tracking of behavioral changes in families with children before and during the COVID-19 pandemic, identifying increased use of some NPIs when ill but no additional illness-associated work or school disruptions.

## HIGHLIGHTS

• Few studies on the pandemic's effect on respiratory illness-related behavior change
• People with respiratory illness stayed home more and avoided contact with others
• The COVID-19 pandemic did not affect illness-associated school or work absenteeism

## INTRODUCTION

Respiratory viral illnesses are associated with societal disruption, including economic and opportunity costs from academic and work productivity loss.^1^, ^2^, ^3^, ^4^ Such costs can be especially high in large outbreaks, as observed during severe influenza epidemics and pandemics and the COVID-19 pandemic.^5^^,^^6^ Studies have examined the mitigating effects of vaccines against these costs of respiratory illnesses^7^^,^^8^ but nonpharmaceutical interventions (NPIs) are not as well studied.

NPIs, such as masking and social distancing, are effective at preventing the transmission of influenza and other viruses,^9^ but success relies on individual engagement in these health behaviors. In studies of NPI use in response to pandemic influenza^10^ and the COVID-19 pandemic,^11^, ^12^, ^13^ participation has been good, often influenced by demographic factors.^14^^,^^15^ How acute respiratory illnesses (ARIs) affect behavior change, both in nonpandemic and pandemic settings, has not been elucidated.

The burden of ARI on children and parents in a household can be high, not only due to individual absenteeism or productivity loss but also because of caregiving responsibilities for working adults.^2^^,^^16^ Households with young children may be most likely to benefit from protective health behaviors considering frequent exposures to respiratory viruses^17^^,^^18^ and higher risks of viral transmission.^19^

The authors assessed the effect of ARIs on work and school participation and NPI use during a typical respiratory viral season, comparing effects among 3 periods from before the COVID-19 pandemic through the first year of the pandemic (pre– and post–COVID-19 vaccine approval). Additionally, the authors evaluated the associations between (1) specific ARI symptoms or case definitions and (2) school and work participation or health behaviors.

## METHODS

### Study Population

Households with school-aged children in King County, Washington, were enrolled in prospective longitudinal cohort studies between November 14, 2019, and June 19, 2021, including an interventional study of home-based testing and treatment for influenza^20^ and an observational study of respiratory viral infections.^18^ Participants were recruited from elementary and middle schools in the Seattle metropolitan region in the fall of 2019 and 2020. Households participating in the 2019–2020 study year were offered continued participation during the 2020–2021 year. Participants were screened using online questionnaires. Eligible households consisted of 3 or more individuals who slept in the home for at least 4 days a week, among whom there was at least 1 adult English speaker and 1 child aged 3 months through 17 years. Eligibility criteria were adapted from a prior household study^17^ to maximize the enrollment of households with children. Research staff conducted informed consent over the phone with an adult from eligible households. All household participants reviewed and provided electronic consent. Parents or guardians provided consent for children aged <18 years and children aged 7–17 years provided electronic assent alongside a parent or guardian's consent. Questionnaires were provided through Project REDCap (Research Electronic Data Capture), a web-based platform.^21^^,^^22^ This study was approved by the University of Washington IRB. The STROBE Statement was used as a reporting guideline.

Statewide shutdowns in Washington began in March 2020, including school closures and the Stay Home, Stay Healthy order for isolation except for essential activities. A timeline of major events related to the COVID-19 pandemic is summarized in Appendix Table 1.

### Measures

Participants completed enrollment questionnaires online. Household reporters were prompted to complete weekly symptom logs on behalf of all participating household members and an illness questionnaire if any participant reported signs or symptoms consistent with ARI, defined as either acute cough or 2 or more concurrent symptoms (see Appendix Table 2 for all qualifying symptoms) with symptom onset within 72 hours (within 48 hours for interventional study participants in 2019–2020).^18^ Participants reporting ARIs completed a follow-up illness questionnaire 7 days following the illness report (up to 10 days from symptom onset). Questions asked if participants engaged in specific health behaviors and if participants experienced work or school impacts in response to illness. For example, a response that a participant “worked from home” because they were ill implied that they did not work from home at the time of the illness. The authors were unable to ascertain baseline behaviors, work, or school activities for each period.

All ARIs with follow-up illness questionnaires were included in this analysis. Symptoms were classified into 3 groups: constitutional (at least 1 sign or symptom among fever, fatigue, muscle or body aches, chills, sweats, and headache), respiratory (at least 1 symptom among runny nose, sore throat, cough, and trouble breathing), or gastrointestinal (nausea or vomiting, diarrhea). COVID-19–like illness 1 (CLI1) was defined as fever with cough or difficulty breathing, using the original Centers for Disease Control and Prevention (CDC) COVID-19 case definition,^23^ and COVID-19–like illness 2 was defined as either 2 of the following symptoms (fever, chills, rigors, myalgia, headache, sore throat, new olfactory and taste disorder[s]) or at least 1 respiratory symptom (cough, shortness of breath, or difficulty breathing).^24^ Influenza-like illness (ILI) was defined as fever with cough and/or sore throat.^25^

### Statistical Analysis

For the analysis, the authors defined 3 periods: pre–COVID-19 pandemic (November 14, 2019–March 22, 2020; Period 1); early, pre–COVID-19 vaccine pandemic (March 23, 2020–December 10, 2020; Period 2); and post–COVID-19 vaccine pandemic (December 11, 2020–June 19, 2021; Period 3). These periods were determined a priori, bookmarked by local and national events of significance: (1) Governor Jay Inslee signed a Stay Home, Stay Healthy emergency proclamation for Washington state on March 23, 2020, a significant event for families in Washington,^26^ and (2) the U.S. Food and Drug Administration issued the first emergency use authorization for a COVID-19 vaccine for individuals 16 years and older on December 11, 2021.^27^

The study restricted school- and work-related analyses to plausible students and working adults by age (5–17 years and ≥18 years, respectively) and occupation, when available. Using generalized estimating equations to account for household clusters and repeated measurements for individuals, the authors compared the frequency of behavior changes and school or work impact in Periods 2 and 3 compared with Period 1. The authors used logistic regression with generalized estimating equations to calculate the ORs of ARI signs and symptoms on reported behaviors and school or work impacts, with adjustments for potential confounders selected a priori by experts in epidemiology, public health, and infectious diseases and supported by the literature: period,^28^^,^^29^ age,^30^^,^^31^ sex,^32^ race and ethnicity,^33^ and household income.^33^^,^^34^ Separate models were developed for CLI, using 2 case definitions, and ILI. For the NPI *covering a cough or sneeze*, the authors excluded cough or runny nose in the model due to a high degree of overlap between the predictor and outcome. The authors defined *p*<0.05 as statistically significant. All data analyses were performed using R, version 4.1.1 (R Foundation for Statistical Computing, Vienna, Austria).^35^

## RESULTS

Of 1,861 participants in 470 households enrolled in the study November 2019–June 2021, 642 (34.5%) unique participants from 310 (66.0%) unique households reported at least 1 ARI and of these, 90.5% of participants (n=581) from 94.5% of households (n=293) completed the 1-week follow-up illness questionnaire (Appendix Table 3). One-week follow-up response rates after ARIs declined throughout the study: from 482/535 (90.1%) of ARIs in Period 1 to 218/264 (82.6%) in Period 2 then 184/234 (78.6%) in Period 3. In this population reporting ARIs with follow-up data, the largest age groups were 18 to 49 years (n=284, 48.9%) and 5 to 12 years (n=161, 27.7%) (Table 1). The majority of individuals reporting ARIs identified as female (n=331, 57.0%), White (n=463, 79.7%), and non-Hispanic (n=541, 93.1%). Most individuals did not report chronic medical conditions (n=413, 71.1%), and adults were highly educated (n=160, 49.8%, with an advanced degree). Of the 293 households, 131 (44.7%) had a child aged under 5 years, 193 (65.9%) had a child aged 5 to 12 years, and 83 (30.4%) had at least 1 child in daycare. The mean household size was 4 (SD=0.8) individuals. For households reporting an annual household income, 222 (75.8%) reported greater than $100,000. In Period 2 (March 23, 2020–December 10, 2020, post–Washington State Stay at Home order), a higher proportion of adults aged 18–49 years (59.9%), females (65.5%), and those reporting 1 or more chronic medical conditions (32.8%) reported ARIs compared with the proportions in Periods 1 and 3. In Period 3 (December 11, 2020–June 19, 2021: post–U.S. Food and Drug Administration Emergency Use Authorization for the Pfizer-BioNTech COVID-19 vaccine) a higher proportion of children <5 years of age (17.2%) and those with no chronic medical conditions (79.8%) reported ARIs. The median duration of study participation for participants reporting ARIs with follow-up data was 75.3 weeks (IQR=43.9).

**Table 1:** Unique Individual and Households With Acute Respiratory Illnesses Before and During the COVID-19 Pandemic

	Period 1 11/14/19–3/22/20 pre–COVID-19 pandemic n (%)	Period 2 3/23/20–12/10/20 early, pre–COVID-19 vaccine pandemic^a^ n (%)	Period 3 12/11/20–6/19/21 post–COVID-19 vaccine pandemic^b^ n (%)	All individuals or households reporting acute respiratory illnesses^c^ n (%)
Characteristic^d^	N=364^e^	N=177^e^	N=163^e^	N=581^f^
Age, years				
Mean (SD)	26.0 (18.6)	29.7 (17.3)	24.9 (18.0)	26.4 (18.4)
<5	40 (11.0%)	22 (12.4%)	28 (17.2%)	70 (12.0%)
5–12	112 (30.8%)	31 (17.5%)	38 (23.3%)	161 (27.7%)
13–17	15 (4.1%)	5 (2.8%)	11 (6.7%)	29 (5.0%)
18–49	171 (47.0%)	106 (59.9%)	81 (49.7%)	284 (48.9%)
≥50	26 (7.1%)	13 (7.3%)	5 (3.1%)	37 (6.4%)
Sex				
Female	206 (56.6%)	116 (65.5%)	93 (57.1%)	331 (57.0%)
Male	157 (43.1%)	60 (33.9%)	70 (42.9%)	249 (42.9%)
Other	1 (0.3%)	1 (0.6%)	0 (0%)	1 (0.2%)
Ethnicity				
Hispanic	25 (6.9%)	15 (8.5%)	12 (7.4%)	40 (6.9%)
Non-Hispanic	339 (93.1%)	162 (91.5%)	151 (92.6%)	541 (93.1%)
Race				
American Indian or Alaska Native	0 (0%)	0 (0%)	2 (1.2%)	2 (0.3%)
Asian	24 (6.6%)	12 (6.8%)	10 (6.1%)	37 (6.4%)
Black	4 (1.1%)	1 (0.6%)	2 (1.2%)	6 (1.0%)
Multiple	35 (9.6%)	13 (7.3%)	11 (6.7%)	53 (9.1%)
Other	6 (1.6%)	6 (3.4%)	4 (2.5%)	13 (2.2%)
White	289 (79.4%)	143 (80.8%)	131 (80.4%)	463 (79.7%)
Missing	6 (1.6%)	2 (1.1%)	3 (1.8%)	7 (1.2%)
Chronic medical conditions				
None	257 (70.6%)	119 (67.2%)	130 (79.8%)	413 (71.1%)
One or more	105 (28.8%)	58 (32.8%)	33 (20.2%)	166 (28.6%)
Prefer not to say	2 (0.5%)	0 (0%)	0 (0%)	2 (0.3%)

Highest education level^g^	N=197^d^	N=129^d^	N=86^d^	N=321^e^

Less than high school or graduated from high school/GED	20 (10.2%)	14 (10.9%)	8 (9.3%)	36 (11.2%)
Some college or bachelor's degree	81 (41.1%)	45 (34.9%)	30 (34.9%)	124 (38.6%)
Advanced degree	96 (48.7%)	60 (46.5%)	48 (55.8%)	160 (49.8%)
Missing	0 (0%)	10 (7.8%)	3 (3.5%)	1 (0.3%)

Household size	N=188^e^	N=123^e^	N=116^e^	N=293^f^

Mean (SD)	4.0 (0.8)	4.0 (0.9)	4.0 (0.9)	4.0 (0.8)
Annual household income, USD				
≤$100,000	31 (16.5%)	15 (12.2%)	15 (12.9%)	43 (14.7%)
>$100,000	138 (73.4%)	99 (80.5%)	90 (77.6%)	222 (75.8%)
Missing	19 (10.1%)	9 (7.3%)	11 (9.5%)	28 (9.6%)
Child in household				
<5 years old	85 (45.2%)	65 (52.8%)	53 (45.7%)	131 (44.7%)
5–12 years old	125 (66.5%)	79 (64.2%)	75 (64.7%)	193 (65.9%)
13–18 years old	40 (21.3%)	23 (18.7%)	24 (20.7%)	65 (22.2%)
Attends daycare	58 (30.9%)	39 (31.7%)	38 (32.8%)	89 (30.4%)

a Following the Washington state “Stay Home, Stay Healthy” emergency proclamation on March 23, 2020.

b Following the U.S. Food and Drug Administration's first emergency use authorization for a COVID-19 vaccine for individuals aged 16 years and older on December 11, 2021.

c Excludes 133 unique participants from 102 unique households with missing follow-up illness questionnaires.

d Participants reported demographic information at study enrollment.

e For participants or households with multiple acute respiratory illnesses in a period, demographic information was only reported once.

f For participants or households with multiple acute respiratory illnesses throughout the study period, demographic information was only reported once and does not reflect the sums of data from each period in this table.

g Only asked for participants aged 18 years and older. GED, general education development (United States high school diploma alternative); USD, United States dollar.

A total of 884 ARIs with follow-up illness data were reported: 482 during Period 1 (November 14, 2019–March 22, 2020, pre–COVID-19 pandemic), 218 during Period 2 (March 23, 2020–December 10, 2020, post–Washington State Stay at Home order), and 184 in Period 3 (December 11, 2020–June 19, 2021, post–U.S. Food and Drug Administration Emergency Use Authorization for the Pfizer-BioNTech COVID-19 vaccine; Appendix Table 4).

Among the 581 participants who reported at least 1 ARI during the study, 472 (81.2%) reported ARI(s) in only 1 period, of which 57.8% reported ARI(s) in Period 1 (Appendix Table 5). Of 95 (16.4%) participants who reported ARIs in 2 periods, 56.8% reported ARIs in Periods 1 and 2. Only 14 (2.4%) of all ARI-reporting participants reported ARIs in all 3 periods. There was a mean of 1.5 (SD=0.9) ARIs reported per individual over the entire study period with a mean of 3.3 (SD=1.4) symptoms reported per ARI. Runny nose was the most frequently reported symptom (n=547 ARIs, 61.9%), followed by fatigue (n=480, 54.3%) and sore throat (n=458, 51.8%) (Table 2). The proportion of ARIs with runny nose (47.2%), fatigue (48.6%), and cough (33.9%) decreased in Period 2 compared with Period 1. Of 884 ARIs, 160 (18.1%) met the ILI case definition. Only 108 (12.2%) met the initial CDC COVID-19 case definition from January 2020,^23^ but with the broader case definition implemented in April 2020, 662 (74.9%) ARIs qualified as CLIs.^24^ More ARIs met one of these case definitions in Period 1.

**Table 2:** Signs and Symptoms Reported in Acute Respiratory Illness Episodes and Acute Respiratory Illnesses Meeting COVID-19-Like-Illness and Influenza-like Illness Case Definitions

Sign or symptom	Period 1 11/14/19–3/22/20 pre–COVID-19 pandemic	Period 2 3/23/20–12/10/20 early, pre–COVID-19 vaccine pandemic^a^	Period 3 12/11/20–6/19/21 post–COVID-19 vaccine pandemic^b^	All acute tespiratory illnesses^c^
	N=482 n (%)	N=218 n (%)	N=184 n (%)	N=884 n (%)
Runny nose	322 (66.8)	103 (47.2)	122 (66.3)	547 (61.9)
Fatigue	278 (57.7)	106 (48.6)	96 (52.2)	480 (54.3)
Sore throat	263 (54.6)	115 (52.8)	80 (43.5)	458 (51.8)
Cough	275 (57.1)	74 (33.9)	59 (32.1)	408 (46.2)
Headache	183 (38.0)	82 (37.6)	72 (39.1)	337 (38.1)
Fever	161 (33.4)	53 (24.3)	41 (22.3)	255 (28.8)
Muscle or body ache	117 (24.3)	37 (17.0)	35 (19.0)	189 (21.4)
Chills	101 (21.0)	31 (14.2)	20 (10.9)	152 (17.2)
Nausea or vomiting	74 (15.4)	41 (18.8)	21 (11.4)	136 (15.4)
Diarrhea	38 (7.9)	26 (11.9)	11 (6.0)	75 (8.5)
Sweats	49 (10.2)	5 (2.3)	9 (4.9)	63 (7.1)
Trouble breathing	26 (5.4)	13 (6.0)	9 (4.9)	48 (5.4)
Ear pain or discharge	20 (4.1)	14 (6.4)	9 (4.9)	43 (4.9)
Rash	3 (0.6)	2 (0.9)	1 (0.5)	6 (0.7)
Syndrome				
COVID-19-like illness 1 (CLI 1) d	86 (17.8)	11 (5.0)	11 (6.0)	108 (12.2)
COVID-19-like illness 2 (CLI 2) e	399 (82.8)	145 (66.5)	118 (64.1)	662 (74.9)
Influenza-like illness (ILI) f	114 (23.7)	32 (14.7)	14 (7.6)	160 (18.1)

a Following the Washington “Stay Home, Stay Healthy” emergency proclamation on March 23, 2020.

b Following the U.S. Food and Drug Administration's first emergency use authorization for a COVID-19 vaccine for individuals aged 16 years and older on December 11, 2021.

c Excludes 149 acute respiratory illnesses with missing follow-up illness questionnaires.

d Per initial Centers for Disease Control and Prevention (CDC) case definition: fever AND symptoms of lower respiratory illness (e.g., cough, shortness of breath).

e Per CDC case definition (April 5, 2020): at least 2 of the following symptoms (fever, chills, rigors, myalgia, headache, sore throat, new olfactory and taste disorder(s)) or at least 1 of the following symptoms (cough, shortness of breath, or difficulty breathing).

f Per CDC case definition: fever AND cough and/or sore throat.

**Figure 1:**
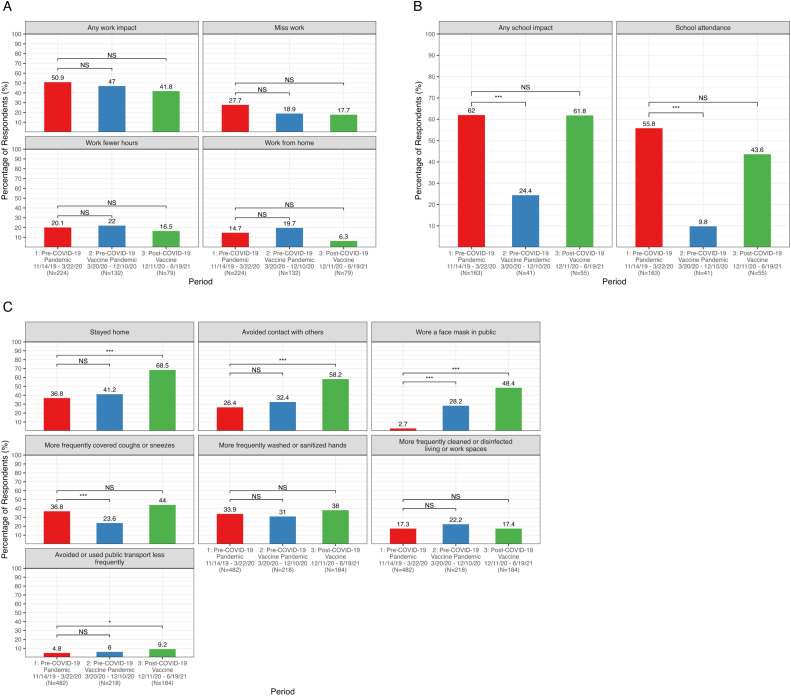
Participant-reported illness-related work (A) and school (B) impacts and changes in health behaviors (C) in the past week due to acute respiratory illness. Frequency plots show the percentage of individuals with acute respiratory illnesses reporting school or work impacts or changes in health behaviors in the week following their illness by period: Period 1 (red): November 14, 2019–March 22, 2020 (pre–COVID-19 pandemic), Period 2 (blue): March 23, 2020–December 10, 2020 (early, pre–COVID-19 vaccine COVID-19 pandemic), and Period 3 (green): December 11, 2020–June 19, 2021 (post–COVID-19 vaccine). Annotated brackets indicate a significant change in percentage (*p<0.05, **p<0.01, ***p<0.001, and NS calculated using Wald's test with GEE, adjusted for household clusters) from Period 1 to Period 2 or 3 for the work or school impact or health behavior. GEE, generalized estimating equations; NS, not significant.

For ill working adults (n=436 ARIs), work disruptions due to illness decreased from prepandemic (Period 1) to both the pre–COVID-19 vaccine (Period 2) and post-vaccine pandemic periods (Period 3): there were small decreases in ARIs impacting work from 50.9% (Period 1) to 41.8% (Period 3, *p*=0.16), working from home from 14.7% (Period 1) to 6.3% (Period 3, *p*=0.06), and missing work from 27.7% (Period 1) to 18.8% (Period 2, *p*=0.06) and 17.7% (Period 3, *p*=0.08) (Figure 1A, Appendix Table 6). The mean (SD) number of workdays missed due to ARI decreased from 2.5 (1.7) days prepandemic (Period 1) to 1.4 (0.6) days in the post-vaccine pandemic (Period 3, *p*=0.58).

**Figure 2:**
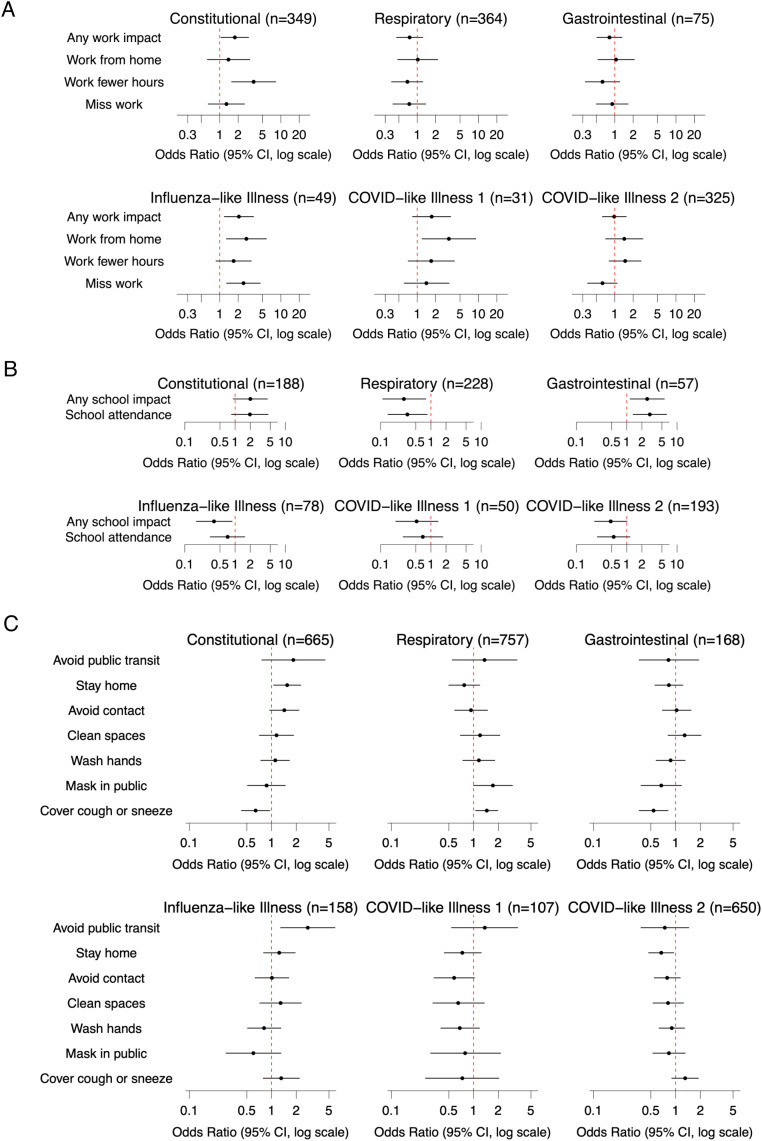
Associations between acute respiratory illness signs or symptoms and impacts on work (A), school (B), and health behaviors (C). Forest plots demonstrate the ORs of specific behaviors or impacts associated with an illness including the symptom or meeting the case definition in question, compared with an illness without the symptom or not meeting the case definition, respectively. Horizontal lines show 95% CIs. Individual models for each impact or behavior were adjusted for period, age group, sex, race/ethnicity, household income, and number of symptoms. Illness was defined per acute respiratory illness case definition: cough or 2 qualifying symptoms (fever, sore throat, runny nose, muscle or body aches, headache, difficulty breathing, fatigue, nausea or vomiting; for participants <18 years of age, ear pain or drainage, rash, and diarrhea were also qualifying symptoms). Constitutional syndrome was defined as at least 1 symptom among fever, fatigue, muscle/body aches, chills, sweats, and headache. Respiratory syndrome was defined as at least 1 symptom among runny nose, sore throat, cough, and trouble breathing. Gastrointestinal syndrome was defined as illnesses including nausea/vomiting and/or diarrhea. Influenza-like illness was defined per CDC case definition: fever and cough and/or sore throat. COVID-like illness 1 was defined per initial CDC COVID-19 case definition: fever AND symptoms of lower respiratory illness (e.g., cough, shortness of breath). COVID-like illness 2 was defined by the following CDC case definition (April 5, 2020): at least 2 of the following symptoms (fever, chills, rigors, myalgia, headache, sore throat, new olfactory and taste disorder[s]) or at least 1 of the following symptoms (cough, shortness of breath, or difficulty breathing). For the health behavior “cover a cough or sneeze,” the authors excluded cough or runny nose in the model due to a high degree of overlap between the predictor and outcome. CDC, Centers for Disease Control and Prevention.

ARIs with constitutional symptoms were associated with increased odds of work disruption compared with ARIs without these symptoms (OR=1.91; 95% CI=1.06, 3.43), specifically working fewer hours (OR=6.63; 95% CI=2.24, 19.6), but not missing work (OR=1.01; 95% CI=0.48, 2.11) or working from home (OR=1.11; 95% CI=0.48, 2.56) (Figure 2A, Appendix Table 7). Compared with non-ILIs, ARIs meeting the ILI case definition were associated with increased odds of affecting work activities (OR=2.72; 95% CI=1.33, 5.58): specifically, missing work (OR=2.22; 95% CI=1.09, 4.55) and working from home (OR=2.63; 95% CI=1.19, 5.84) but not working fewer hours (OR=1.82; 95% CI=0.89, 3.72). ARIs meeting the initial CLI case definition were associated with increased odds of working from home (OR=3.45; 95% CI=1.23, 9.71) compared with non-CLI ARIs, without any general impacts to work (OR=2.15; 95% CI=0.83, 5.55). ARIs with gastrointestinal symptoms or meeting the later CLI case definition did not have significant associations with work.

For ill school-aged children (n=259 ARIs), there was a drop in school disruptions and absenteeism from prepandemic (Period 1) to the pre-vaccine pandemic (Period 2): the percentage of ARIs impacting school activities decreased from 62.0% to 24.4% (*p*<0.001) and impacts to class attendance decreased from 55.8% to 9.8% (*p*<0.001). (Figure 1B, Appendix Table 6). Between the prepandemic (Period 1) and the post-vaccine pandemic (Period 3) periods, there were comparable disruptions to school activities (62.0% vs 61.8%, *p*=0.98) and class attendance (55.8% vs 43.6%, *p*=0.12). Among ARIs associated with school absenteeism, the mean (SD) number of days missed did not change substantially over time: 2.3 (1.4) in Period 1, 2.4 (1.8) in Period 2, and 2.3 (1.8) in Period 3; *p*=0.90 and *p*=0.97 for Period 1 vs Period 2 and Period 1 vs Period 3, respectively.

Only ARIs with gastrointestinal symptoms were associated with higher odds of school disruptions (OR=2.55; 95% CI=1.17, 5.57; Figure 2B, Appendix Table 8) and higher odds of missing class (OR=2.88; 95% CI=1.34, 6.18) compared with those without gastrointestinal symptoms. Respiratory symptoms appeared to have a protective association with ORs of 0.29 (95% CI=0.11, 0.79) for any school impacts and 0.34 (95% CI=0.14, 0.84) for school absenteeism. ARIs meeting the ILI case definition also was associated with decreased odds of any school disruption (OR=0.38; 95% CI=0.17, 0.87). ARIs with constitutional symptoms or meeting either CLI case definition did not have any clear associations with school impacts.

The percentage of ARIs associated with changes in NPI use ranged from minimal use of facemasks in public (2.7% ARIs) prepandemic (Period 1) to 68.5% ARIs associated with staying at home after COVID-19 vaccine authorization (Period 2, Figure 1C, Appendix Table 6). Masking in public in response to ARIs increased after the COVID-19 pandemic began from 2.7% (Period 1) to 28.0% (Period 2, *p*<0.001) and again after the COVID-19 vaccine authorization to 48.4% (Period 3, *p*<0.001, compared with prepandemic, Period 1). Fewer individuals with ARIs covered coughs or sneezes during the early pandemic period compared with prepandemic (24.3% Period 2 vs 36.9% Period 1, *p*<0.001). After COVID-19 vaccine authorization, more individuals with ARIs stayed home (68.5% Period 3 vs 36.7% Period 1, *p*<0.001) and avoided contact with non-household members (58.2% Period 3 vs 26.3% Period 1, *p*<0.001) compared with during the prepandemic period.

ARIs with constitutional symptoms were associated with higher odds of staying home (OR=1.55; 95% CI=1.06, 2.27) compared with ARIs without constitutional symptoms (Figure 2C, Appendix Table 9). ARIs meeting the ILI case definition had higher odds of decreased use or avoidance of public transportation (OR=2.76; 95% CI=1.29, 5.91) compared with non-ILIs. ARIs with respiratory symptoms were associated with higher odds of covering coughs or sneezes more frequently (OR=1.45; 95% CI=1.06, 1.98) compared with those without. There were 3 negative associations: ARIs with constitutional or gastrointestinal symptoms were associated with decreased odds of covering coughs and sneezes (OR=0.64; 95% CI=0.43, 0.95, and OR=0.54; 95% CI=0.36, 0.81, respectively) compared with ARIs without each set of symptoms, and ARIs meeting the second, broader CLI case definition were associated with decreased odds of staying home (OR=0.67; 95% CI=0.47, 0.95) compared with non-CLI ARIs. ARIs meeting the early CLI case definition did not have any substantial associations with behavior change, and no syndrome or case definition had substantial associations with avoiding contact with non-household members, masking in public, or more frequently cleaning or disinfecting living or workspaces.

## DISCUSSION

Using longitudinal participant-reported illness and behavior data, this study investigated changes in school-, work-, and health-related behaviors following ARIs and the associations between ARIs, comparing ARIs with specific symptoms to those without, and these behaviors among households in the Seattle metropolitan area from November 2019 to June 2021. To the authors’ knowledge, this is one of the first studies to compare illness-related behavior changes in families with young children before and during the COVID-19 pandemic. The study found that for most illnesses during the first year of the pandemic, compared with prepandemic illnesses, participants reported increased NPI use—specifically, staying home, avoiding contact with non-household members, and masking in public—but did not report increased work or school disruptions. Additionally, the authors found some associations among symptom groups, NPI use, and work and school disruptions.

The study's prepandemic data provided a unique snapshot of illness-related NPI use during a typical respiratory viral season. Of 7 studied NPIs, 4—staying home, avoiding contact with non-household members, masking in public, and avoiding or reducing public transportation use—increased from prepandemic levels and were the most reported NPIs during illness. Of note, only masking increased during the early, pre–COVID-19 vaccine pandemic. Masking started as an uncommon practice prepandemic and its adoption during the early pandemic likely resulted from early statewide mask directives and increasing mask availability. The use of all 3 NPIs increased after the COVID-19 vaccine authorization in December 2020. Other U.S.-based studies, which focused on NPI use outside of illness, reported high levels of social distancing early in the pandemic which declined over time.^36^, ^37^, ^38^ The delayed uptake of staying home, avoiding contact outside the household, and limiting public transportation use when ill may have resulted from initial decreased perceived risk,^31^ increased familiarity with these NPIs over time,^39^^,^^40^ or increased time outside of the home or with non-household members at baseline in the reopening phases, allowing for a measurable difference in behavior when ill. The decrease in covering coughs or sneezes in the early pandemic may have been an artifact of fewer ARIs with runny nose or cough or may have been related to engagement in other behaviors (e.g., staying home per the Stay Home state order or increased masking), which may have reduced societal pressure to cover coughs or sneezes.

Prior studies, primarily focused on influenza, have highlighted the negative impact of illnesses on productivity.^1^, ^2^, ^3^^,^^41^^,^^42^ This study's estimates of workdays lost were consistent with prior reports,^3^^,^^16^ but percentages of individuals missing work due to illness before or during the COVID-19 pandemic were on the lower end of the ranges reported (20%–75%) in studies on influenza or ILI.^16^ The population may have transitioned to full-time remote work early which may have reduced the impact of ARIs on productivity. The eligibility requirement for internet access may have also been selected for workers less likely to be impacted by telework. By virtue of the study design, the authors may have captured milder ARIs that were less likely to impact work: only 18.1% of ARIs met ILI criteria.

This study's estimates of school days missed due to ARI were comparable to those reported during peak influenza seasons.^43^ The decreased impact of ARIs on school activities and class attendance during the early COVID-19 pandemic when schools were fully remote in King County suggests that remote education may have been a mitigation factor. However, though ARI impacts on school activities and attendance never exceeded prepandemic levels during the COVID-19 pandemic periods, published reports of learning losses and chronic absenteeism suggest that the pandemic had lasting impacts on students beyond these metrics.^44^^,^^45^

The authors found variable associations among ARIs with grouped symptoms or ARIs meeting case definitions and the outcomes of interest. For NPI use, the most substantial association was between ILIs and decreased use of public transportation. Constitutional symptoms and ILIs were associated with both general and specific work impacts. Only ARIs with gastrointestinal symptoms had substantial negative associations with school impacts. This heterogeneity suggests that symptomatology may not easily identify ARIs most likely to impact behavior, school, or work, but more research into the correlation between these symptoms and illness severity is needed to further understand these relationships.

This study had several unique strengths: foremost, the remote study design allowed for uninterrupted study continuation with standardized weekly symptom surveys through the COVID-19 pandemic and afforded the comparison of behaviors and impacts during a typical respiratory season versus during the pandemic, when participants may have been on heightened alert for ARI symptoms. Additionally, the prospective design with weekly follow-up after an ARI allowed for the capture of illness-related NPI use and effects on school and work as well as measurement of the association between symptoms and these outcomes. Though the authors used participant-reported data, the short period of time (7 days) between the ARI report and follow-up reduced the risk of recall bias. Lastly, the study focused on ARIs in households, including many with young children, as they play significant roles in respiratory viral transmission.^46^ Researchers have considered using households in respiratory viral surveillance as some symptoms may be early sentinels for respiratory viral seasons^47^ and school absenteeism may be used in ILI surveillance.^48^^,^^49^

### Limitations

Limitations included the observational nature of this study and small sample sizes for some variables in these models. Though the study aimed to be inclusive with recruitment, the cohort was not representative of the Seattle metropolitan area or U.S. households. For example, three fourths of participants reported income greater than or equal to $100,000 in comparison to the median household income of $106,326 in King County from 2017–2021.^50^ The study cohort was also highly educated. The focus on households with children resulted in lower enrollment of individuals aged 50 years or older. Generalizability may also be limited by local policy and contextual factors, which may make the findings specific to this region, and apart from time, the study was unable to adjust for pandemic-related factors (e.g., the number of COVID-19 cases in the community and the availability of point-of-care COVID-19 tests).The authors did not ascertain school or work attendance or use of nonpharmaceutical interventions when well. The authors were unable to rule out nonresponse bias for the participants who did not complete illness follow-up surveys. Though participant and ARI characteristics did not appear to differ greatly, the authors were unable to determine if other markers of illness severity aside from types or number of symptoms were contributing factors. The authors were limited to participants’ interpretations of survey questions and responses may have been affected by social desirability bias. Lastly, this analysis did not distinguish between ARIs with and without positive viral testing.

## CONCLUSIONS

In conclusion, the uninterrupted remote study of ARIs in households with children before and after the start of the COVID-19 pandemic offered novel comparisons of illness-related school and work impacts and NPI usage over 2 years. After the start of the COVID-19 pandemic, there was increased NPI use (e.g., staying home, masking in public, reducing contact with non-household members, and limiting public transportation) but no increased work or school disruptions. Future longitudinal studies in households would help correlate changes in behavior to respiratory virus transmission and determine how policymakers can invoke greater NPI use in future epidemics and pandemics.

## Appendix Supplementary materials

Image, application 1

## Footnotes

Supplementary material associated with this article can be found in the online version at doi:10.1016/j.focus.2024.100248.
