# Supplementary material for: Absenteeism and Health Behavior Trends Associated With Acute Respiratory Illness Before and During the COVID-19 Pandemic in a Community Household Cohort, King County, Washington
Source: AJPM Focus. 2024 Jun 6;3(4):100248. doi: 10.1016/j.focus.2024.100248 (PMC11264170; doi:10.1016/j.focus.2024.100248)
Supplement: Supplementary file 1 [file mmc1.docx]

**Appendix Table of Contents**

**Appendix Table 1.**Timeline of Major COVID-19 Pandemic Related Events in King County, WA

**Appendix Table 2.**List of signs or symptoms used in household study to determine for eligibility for nasal swab specimen collection

**Appendix Table 3.** Study Participant Flowchart

**Appendix Table 4.** Individual and Household Characteristics Associated by Unique Acute Respiratory Illness Episode Before and During the SARS-CoV-2 Pandemic

**Appendix Table 5.** Distribution of Participants Reporting Acute Respiratory Illnesses by Study Periods

**Appendix Table 6.** Health Behavior Changes and School or Work Impacts Reported by Participants due to Acute Respiratory Illness by Acute Respiratory Illness Episode

**Appendix Table 7.** Odds Ratios for Work Impacts by Acute Respiratory Illness Symptom or Syndrome Case Definition

**Appendix Table 8.** Odds Ratios for School Impacts by Acute Respiratory Illness Symptom or Syndrome Case Definition

**Appendix Table 9.** Odds Ratios for Changes in Health Behaviors by Acute Respiratory Illness Symptom or Syndrome Case Definition

**Appendix Table 1.**Timeline of Major COVID-19 Pandemic Related Events in King County, WA

| **Date** | **Event** |
| --- | --- |
| March 13, 2020 | Statewide school closures |
| March 23, 2020 | Governor Inslee signed a “Stay Home, Stay Healthy” emergency proclamation for Washington state |
| May 31, 2020 | “Stay Home, Stay Healthy” emergency proclamation expires; King County enters Phase 1 of Washington’s Safe Start plan |
| May 18, 2020 | King County directive signed urging face coverings in indoor public places |
| June 5, 2020 | King County approved for a modified Phase 1 of the Governor’s Safe Start plan |
| June 19, 2020 | King County entered Phase 2 of Washington’s Safe Start plan |
| April 5-19, 2021 | Seattle Public Schools district resumes in-person education for all students |
| May 20, 2021 | Directive urging face coverings in indoor public places renewed |

Sources:

1. King County Department of Community and Human Services. COVID-19 Response: A Year in Review Timeline. https://kingcounty.gov/en/legacy/depts/community-human-services/covid/-/media/depts/community-human-services/department/documents/DCHS_COVID-19_Response_Timeline_3-24-2021.ashx?la=en&hash=2696A7041807B07E4640762117208C4C

2. King County Executive Office. COVID Timeline: Restrictions. https://kingcounty.gov/~/media/elected/executive/constantine/initiatives/covid/COVID_Timeline_Restrictions.ashx?la=en

3. King County Executive Office. COVID-19 Response. https://kingcounty.gov/en/legacy/elected/executive/constantine/covid-response/covid

4. OSPI School Facility ReOpening Survey. State of Washington. Published June 9, 2021. https://data.wa.gov/dataset/School-Facility-ReOpening-Survey/9i5d-c2m8/about_data

**Appendix Table 2.**List of signs or symptoms used in household study to determine for eligibility for nasal swab specimen collection

| Acute cough (sufficient on its own) ^a^ | Feeling feverish ^a^ |
| --- | --- |
| Sore throat ^a^ | Runny/stuffy nose ^a^ |
| Muscle or body aches ^a^ | Chills or shivering ^c^ |
| Headache ^a^ | Difficulty breathing ^a^ |
| Fatigue ^a^ | Nausea or vomiting ^a^ |
| Ear pain or ear drainage ^b^ | Rash ^b^ |
| Sweats ^c^ | Diarrhea ^b^ |
| Loss of taste or smell ^e^ |  |

**^a^** Qualifying signs or symptoms included in the acute respiratory illness (ARI) case definition for participants of any age. Two qualifying signs or symptoms were required for case definition of ARI except for cough, which was sufficient by itself.
^b^Qualifying ARI signs or symptoms for participants < 18 years of age
^c^ Included in sign and symptom questions but not a qualifying sign or symptom included in ARI case definition 
^e^ Added to sign and symptom list in April 2020. Not a qualifying sign or symptom included in the ARI case definition for participants of any age.

**Appendix Table 3.** Study Participant Flowchart

**
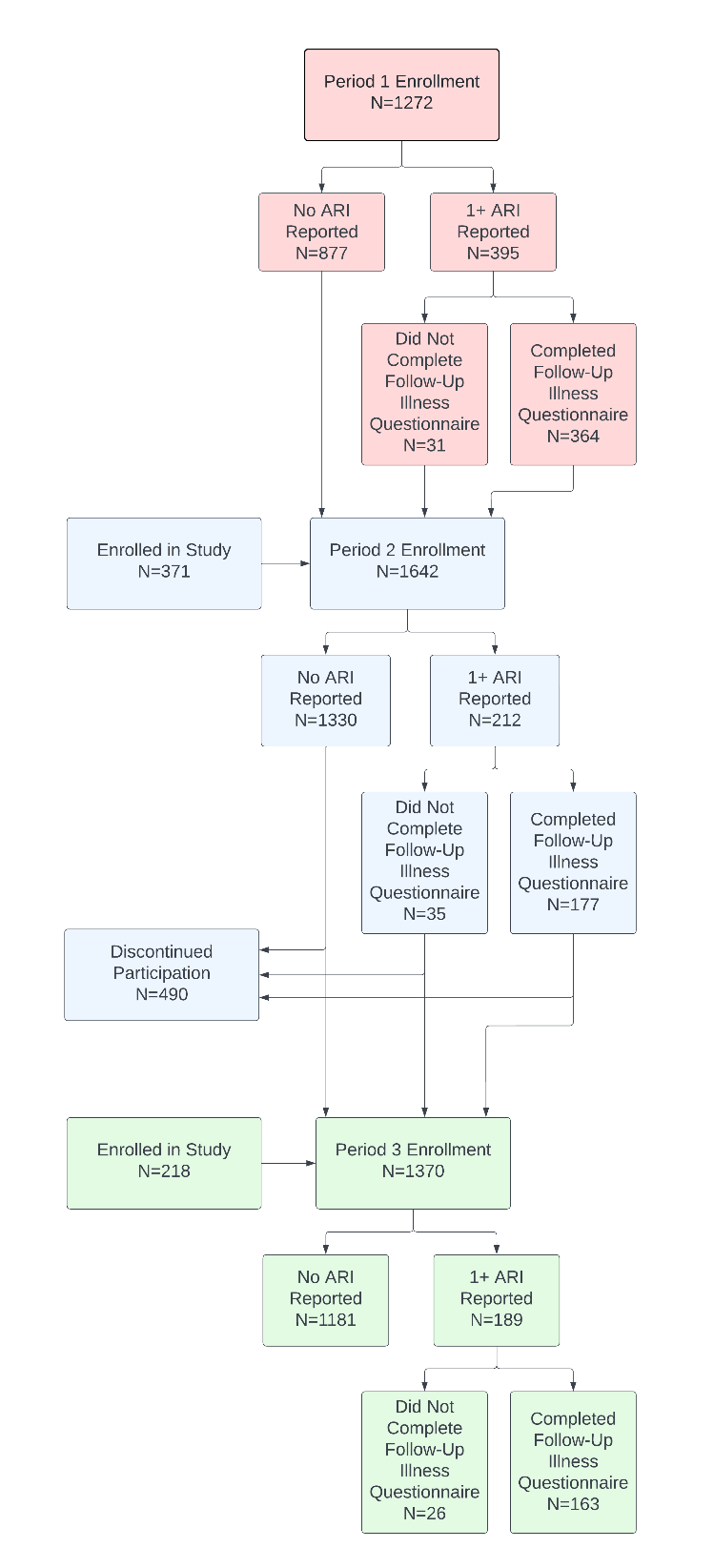
**

**Abbreviations:** ARI, acute respiratory illness; Period 1, Pre- COVID-19 pandemic (11/14/19 – 3/22/20); Period 2, Early, pre-COVID-19 vaccine pandemic, following the Washington state “Stay Home, Stay Healthy” emergency proclamation on March 23, 2020 **(**3/23/20 – 12/10/20); Period 3**,** Post-COVID-19 vaccine EUA pandemic, following the U.S. Food and Drug Administration’s first emergency use authorization for a COVID-19 vaccine for individuals 16 years and older on December 11, 2021 **(**12/11/20 – 6/19/21)

**Appendix Table 4.** Individual and Household Characteristics Associated by Unique Acute Respiratory Illness Episode Before and During the SARS-CoV-2 Pandemic

|  | Period 1  11/14/19 – 3/22/20  Pre- COVID-19 pandemic | Period 2  3/23/20 – 12/10/20 Early, pre-COVID-19 vaccine pandemic^a^ | Period 3  12/11/20 – 6/19/21  Post-COVID-19 vaccine EUA pandemic^b^ | Overall |
| --- | --- | --- | --- | --- |
| Characteristic^c^ | **N=482^d^** | **N=218^d^** | **N=184^d^** | **N=884^e^** |
| Age, years |  |  |  |  |
| Mean (SD) | 25.5 (18.3) | 30.3 (16.9) | 24.3 (18.0) | 26.4 (18.0) |
| < 5 | 60 (12.4%) | 25 (11.5%) | 37 (20.1%) | 122 (13.8%) |
| 5-12 | 145 (30.1%) | 36 (16.5%) | 39 (21.2%) | 220 (24.9%) |
| 13-17 | 17 (3.5%) | 5 (2.3%) | 13 (7.1%) | 35 (4.0%) |
| 18-49 | 232 (48.1%) | 135 (61.9%) | 90 (48.9%) | 457 (51.7%) |
| > 50 | 28 (5.8%) | 17 (7.8%) | 5 (2.7%) | 50 (5.7%) |
| Sex |  |  |  |  |
| Female | 282 (58.5%) | 148 (67.9%) | 104 (56.5%) | 534 (60.4%) |
| Male | 197 (40.9%) | 67 (30.7%) | 80 (43.5%) | 344 (38.9%) |
| Other | 3 (0.6%) | 3 (1.4%) | 0 (0%) | 6 (0.7%) |
| Ethnicity |  |  |  |  |
| Hispanic | 33 (6.8%) | 17 (7.8%) | 15 (8.2%) | 65 (7.4%) |
| Non-Hispanic | 449 (93.2%) | 201 (92.2%) | 169 (91.8%) | 819 (92.6%) |
| Race |  |  |  |  |
| White | 390 (80.9%) | 178 (81.7%) | 147 (79.9%) | 715 (80.9%) |
| Asian | 31 (6.4%) | 13 (6.0%) | 10 (5.4%) | 54 (6.1%) |
| Black | 5 (1.0%) | 1 (0.5%) | 2 (1.1%) | 8 (0.9%) |
| American Indian or Alaska Native | 0 (0%) | 0 (0%) | 2 (1.1%) | 2 (0.2%) |
| Other | 7 (1.5%) | 6 (2.8%) | 6 (3.3%) | 19 (2.1%) |
| Multiple | 42 (8.7%) | 17 (7.8%) | 14 (7.6%) | 73 (8.3%) |
| Missing | 7 (1.5%) | 3 (1.4%) | 3 (1.6%) | 13 (1.5%) |
| Chronic Medical Conditions | | |  |  |
| None | 342 (71.0%) | 144 (66.1%) | 146 (79.3%) | 632 (71.5%) |
| One or more | 137 (28.4%) | 74 (33.9%) | 38 (20.7%) | 249 (28.2%) |
| Prefer not to say | 3 (0.6%) | 0 (0%) | 0 (0%) | 3 (0.3%) |
|  | |  |  |  |
| Highest Education Level^f^ | **N=260** | **N=152** | **N=95** | **N=507** |
| Less than high school or graduated from high school/GED | 4 (1.5%) | 2 (1.3%) | 3 (3.2%) | 9 (1.8%) |
| Some college | 22 (8.5%) | 13 (8.6%) | 9 (9.5%) | 44 (8.7%) |
| Bachelor's degree | 110 (42.3%) | 61 (40.1%) | 30 (31.6%) | 201 (39.6%) |
| Advanced degree | 124 (47.7%) | 76 (50.0%) | 53 (55.8%) | 253 (49.9%) |
| Household Size | |  |  |  |
| Mean (SD) | 4.1 (0.8) | 4.1 (1.0) | 4.0 (1.0) | 4.1 (0.9) |
| Annual Household Income, USD | | |  |  |
| < $100,000 | 81 (16.8%) | 34 (15.6%) | 29 (15.8%) | 144 (16.3%) |
| > $100,000 | 357 (74.1%) | 169 (77.5%) | 140 (76.1%) | 666 (75.3%) |
| Missing | 44 (9.1%) | 15 (6.9%) | 15 (8.2%) | 74 (8.4%) |
| Children in Household | |  |  |  |
| Child < 5 years old | 256 (53.1%) | 125 (57.3%) | 94 (51.1%) | 475 (53.7%) |
| Child 5-12 years old | 330 (68.5%) | 135 (61.9%) | 120 (65.2%) | 585 (66.2%) |
| Child 13-18 years old | 72 (14.9%) | 35 (16.1%) | 38 (20.7%) | 145 (16.4%) |
| Child in daycare | 159 (33.0%) | 78 (35.8%) | 62 (33.7%) | 299 (33.8%) |

Abbreviations: SD, standard deviation; GED, General Education Development (United States high school diploma alternative); USD, United States dollar

^a^ Following the Washington state “Stay Home, Stay Healthy” emergency proclamation on March 23, 2020

**^b^** Following the U.S. Food and Drug Administration’s first emergency use authorization for a COVID-19 vaccine for individuals 16 years and older on December 11, 2021

^c^ Participants reported demographic information at study enrollment

^d^ For participants or households with multiple acute respiratory illnesses in a period, demographic information was repeated in this table for each acute respiratory illness

^e^ For participants or households with multiple acute respiratory illnesses throughout the study period, demographic information was repeated in this table for each acute respiratory illness

^f^ Only asked for participants 18 years and older

**Appendix Table 5.** Distribution of Participants Reporting Acute Respiratory Illnesses by Study Periods

|  | Unique Participants Reporting > 1 Acute Respiratory Illness (ARI) | | |
| --- | --- | --- | --- |
| Periods with ARI(s) Reported | **One Period** | **Two Periods** | **Three Periods** |
|  | **N=472** | **N=95** | **N=14** |
| Period 1^a^ Only | 273 (57.8%) | 0 (0%) | 0 (0%) |
| Period 2^b^ Only | 91 (19.3%) | 0 (0%) | 0 (0%) |
| Period 3^c^ Only | 108 (22.9%) | 0 (0%) | 0 (0%) |
| Periods 1 and 2 | 0 (0%) | 54 (56.8%) | 0 (0%) |
| Periods 2 and 3 | 0 (0%) | 18 (18.9%) | 0 (0%) |
| Periods 1 and 3 | 0 (0%) | 23 (24.2%) | 0 (0%) |
| All Periods | 0 (0%) | 0 (0%) | 14 (100%) |

^a^ Period 1 (11/14/19 – 3/22/20): Pre- COVID-19 pandemic ^b^ Period 2 **(**3/23/20 – 12/10/20): Early, pre-COVID-19 vaccine pandemic; following the Washington state “Stay Home, Stay Healthy” emergency proclamation on March 23, 2020

^c^ Period 3 **(**12/11/20 – 6/19/21)**:** Post-COVID-19 vaccine EUA pandemic; following the U.S. Food and Drug Administration’s first emergency use authorization for a COVID-19 vaccine for individuals 16 years and older on December 11, 2021

**Appendix Table 6.** Health Behavior Changes and School or Work Impacts Reported by Participants due to Acute Respiratory Illness by Acute Respiratory Illness Episode

|  | Period 1  11/14/19 – 3/22/20  Pre- COVID-19 pandemic | Period 2 3/23/20 – 12/10/20 Early, pre-COVID-19 vaccine pandemic | Period 3 12/11/20 – 6/19/21  Post-COVID-19 vaccine pandemic | Overall | P-values | |
| --- | --- | --- | --- | --- | --- | --- |
|  | **N=482** | **N=218** | **N=184** | **N=884** | **Period 1 vs Period 2** | **Period 1 vs Period 3** |
| Health Behavior Changes |  |  |  |  |  |  |
| Stayed home | 177 (36.7%) | 91 (41.7%) | 126 (68.5%) | 394 (44.6%) | 0.21 | <0.001*** |
| Avoided contact with others | 127 (26.3%) | 71 (32.6%) | 107 (58.2%) | 305 (34.5%) | 0.09 | <0.001*** |
| Wore a face mask in public | 13 (2.7%) | 61 (28.0%) | 89 (48.4%) | 163 (18.4%) | <0.001*** | <0.001*** |
| More frequently covered cough/sneezes | 178 (36.9%) | 53 (24.3%) | 81 (44.0%) | 312 (35.3%) | <0.001*** | 0.09 |
| More frequently washed/sanitized hands | 163 (33.8%) | 69 (31.7%) | 70 (38.0%) | 302 (34.2%) | 0.57 | 0.31 |
| More frequently cleaned/disinfected living/work spaces | 83 (17.2%) | 50 (22.9%) | 32 (17.4%) | 165 (18.7%) | 0.08 | 0.96 |
| Avoided/used public transit less frequently | 23 (4.8%) | 15 (6.9%) | 17 (9.2%) | 55 (6.2%) | 0.26 | 0.03* |
| School Impacts^a^ | **N=163** | **N=41** | **N=55** | **N=259** |  |  |
| Any impact to school | 101 (62.0%) | 10 (24.4%) | 34 (61.8%) | 145 (56.0%) | <0.001*** | 0.98 |
| Class attendance | 91 (55.8%) | 4 (9.8%) | 24 (43.6%) | 119 (45.9%) | <0.001*** | 0.12 |
| Work Impacts^b^ | **N=224** | **N=133** | **N=79** | **N=436** |  |  |
| Any impact to work | 114 (50.9%) | 63 (47.4%) | 33 (41.8%) | 210 (48.2%) | 0.52 | 0.16 |
| Missed work | 62 (27.7%) | 25 (18.8%) | 14 (17.7%) | 101 (23.2%) | 0.06 | 0.08 |
| Worked fewer hours | 45 (20.1%) | 29 (21.8%) | 13 (16.5%) | 87 (20.0%) | 0.70 | 0.48 |
| Worked from home | 33 (14.7%) | 27 (20.3%) | 5 (6.3%) | 65 (14.9%) | 0.17 | 0.06 |
| Days of school missed^a^ | **N=91** | **N=4** | **N=24** | **N=119** |  |  |
| Mean (SD) | 2.3 (1.4) | 2.4 (1.8) | 2.3 (1.8) | 2.3 (1.5) | 0.90 | 0.97 |
| Days of work missed^b^ | **N=62** | **N=25** | **N=14** | **N=101** |  |  |
| Mean (SD) | 2.5 (1.7) | 2.3 (1.4) | 1.4 (0.6) | 2.3 (1.6) | 0.58 | <0.001*** |

P-values: **P*<.05, ***P*<.01, ****P*<.001, calculated using Wald’s test with GEE, adjusted for household clusters

^a^ Limited to students by age (5-17 years)
^b^Limited to working adults by age (>18 years) and specified occupation, when available

**Appendix Table 7.** Odds Ratios for Work Impacts by Acute Respiratory Illness Symptom or Syndrome Case Definition

| Predictor: Symptom or Case Definition (n)^a^ | Work Impact | Odds Ratio (95% CI) |
| --- | --- | --- |
| Constitutional (n=349)^b^ | Any impact to work | 1.91 (1.06, 3.43) |
|  | Missed work | 1.01 (0.48, 2.11) |
|  | Worked from home | 1.11 (0.48, 2.56) |
|  | Worked fewer hours | 6.63 (2.24, 19.60) |
| Respiratory (n = 364)^c^ | Any impact to work | 0.64 (0.36, 1.12) |
|  | Missed work | 0.70 (0.36, 1.37) |
|  | Worked from home | 0.96 (0.44, 2.1) |
|  | Worked fewer hours | 0.62 (0.33, 1.16) |
| Gastrointestinal (n = 75)^d^ | Any impact to work | 0.80 (0.46, 1.39) |
|  | Missed work | 0.90 (0.48, 1.71) |
|  | Worked from home | 1.21 (0.60, 2.48) |
|  | Worked fewer hours | 0.68 (0.33, 1.41) |
| Influenza-like illness, ILI (n = 48)^e^ | Any impact to work | 2.72 (1.33, 5.58) |
|  | Missed work | 2.22 (1.09, 4.55) |
|  | Worked from home | 2.63 (1.19, 5.84) |
|  | Worked fewer hours | 1.82 (0.89, 3.72) |
| COVID-19-like illness, CLI 1 (n = 30)^f^ | Any impact to work | 2.15 (0.83, 5.55) |
|  | Missed work | 1.38 (0.56, 3.37) |
|  | Worked from home | 3.45 (1.23, 9.71) |
|  | Worked fewer hours | 1.58 (0.57, 4.42) |
| COVID-19-like illness, CLI 2 (n = 324)^e^ | Any impact to work | 0.99 (0.6, 1.63) |
|  | Missed work | 0.59 (0.32, 1.1) |
|  | Worked from home | 1.45 (0.7, 3.0) |
|  | Worked fewer hours | 1.54 (0.81, 2.94) |

^a^Limited to working adults by age (>18 years) and specified occupation, when available.

Illness was defined per Acute Respiratory Illness (ARI) case definition: cough or two qualifying symptoms (fever, sore throat, runny nose, muscle or body aches, headache, difficulty breathing, fatigue, nausea or vomiting).

^b^ Constitutional syndrome was defined as at least one symptom among fever, fatigue, muscle/body aches, chills, sweats, and headache.

^c^Respiratory syndrome was defined as at least one symptom among runny nose, sore throat, cough, and trouble breathing.

^d^Gastrointestinal syndrome was defined as illnesses including nausea/vomiting and/or diarrhea. ^e^Influenza-like illness was defined per Centers for Disease Control and Prevention (CDC) case definition: fever AND cough and/or sore throat.

^f^COVID-like illness 1 was defined per initial CDC case definition: fever AND symptoms of lower respiratory illness (e.g., cough, shortness of breath)^.^

^g^COVID-like illness 2 was defined by the following CDC case definition (April 5, 2020): at least two of the following symptoms (fever, chills, rigors, myalgia, headache, sore throat, new olfactory and taste disorder(s)) or at least one of the following symptoms (cough, shortness of breath, or difficulty breathing).**Appendix Table 8.** Odds Ratios for School Impacts by Acute Respiratory Illness Symptom or Syndrome Case Definition

| Predictor: Symptom or Case Definition (n)^a^ | School Impact | Odds Ratio (95% CI) |
| --- | --- | --- |
| Constitutional^b^  (n = 188) | Any impact to school | 2.01 (0.92, 4.39) |
|  | Missed class | 1.97 (0.85, 4.52) |
| Respiratory^c^  (n = 228) | Any impact to school | 0.29 (0.11, 0.79) |
|  | Missed class | 0.34 (0.14, 0.84) |
| Gastrointestinal^d^  (n = 57) | Any impact to school | 2.55 (1.17, 5.57) |
|  | Missed class | 2.88 (1.34, 6.18) |
| Influenza-like illness, ILI^e^ (n = 78) | Any impact to school | 0.38 (0.17, 0.87) |
|  | Missed class | 0.71 (0.32, 1.55) |
| COVID-19-like illness, CLI 1^f^  (n = 50) | Any impact to school | 0.52 (0.20, 1.38) |
|  | Missed class | 0.69 (0.28, 1.73) |
| COVID-19-like illness, CLI 2^g^  (n = 193) | Any impact to school | 0.48 (0.23, 1.01) |
|  | Missed class | 0.55 (0.26, 1.16) |

^a^ Limited to students by age (5-17 years). Illness was defined per acute respiratory illness case definition: cough or two qualifying symptoms (fever, sore throat, runny nose, muscle or body aches, headache, difficulty breathing, fatigue, nausea, or vomiting; for participants < 18 years of age, ear pain or drainage, rash, and diarrhea were also qualifying symptoms).

^b^ Constitutional syndrome was defined as at least one symptom among fever, fatigue, muscle/body aches, chills, sweats, and headache.

^c^Respiratory syndrome was defined as at least one symptom among runny nose, sore throat, cough, and trouble breathing.

^d^Gastrointestinal syndrome was defined as illnesses including nausea/vomiting and/or diarrhea. ^e^Influenza-like illness was defined per Centers for Disease Control and Prevention (CDC) case definition: fever AND cough and/or sore throat. ^f^COVID-like illness 1 was defined per initial CDC case definition: fever AND symptoms of lower respiratory illness (e.g., cough, shortness of breath)^.^

^g^COVID-like illness 2 was defined by the following CDC case definition (April 5, 2020): at least two of the following symptoms (fever, chills, rigors, myalgia, headache, sore throat, new olfactory and taste disorder(s)) or at least one of the following symptoms (cough, shortness of breath, or difficulty breathing).

**Appendix Table 9.** Odds Ratios for Changes in Health Behaviors by Acute Respiratory Illness Symptom or Syndrome Case Definition

| Predictor: Symptom or Case Definition (n)^a^ | Health Behavior | Odds Ratio (95% CI) |
| --- | --- | --- |
| Constitutional^b^  (n = 665) | Stayed home | 1.55 (1.06, 2.27) |
|  | Avoided contact with others outside of the household | 1.43 (0.95, 2.15) |
|  | Wore a face mask in public | 0.87 (0.51, 1.47) |
|  | Covered cough or sneeze more frequently | 0.64 (0.43, 0.95) |
|  | Washed or sanitized hands more frequently | 1.11 (0.74, 1.65) |
|  | Cleaned or disinfected living or work spaces more frequently | 1.15 (0.71, 1.85) |
|  | Avoided or decreased public transportation use | 1.84 (0.76, 4.48) |
| Respiratory^c^  (n = 757) | Stayed home | 0.77 (0.50, 1.19) |
|  | Avoided contact with others outside of the household | 0.93 (0.59, 1.48) |
|  | Wore a face mask in public | 1.72 (1.00, 2.97) |
|  | Covered cough or sneeze more frequently | 1.45 (1.06, 1.98) |
|  | Washed or sanitized hands more frequently | 1.16 (0.74, 1.81) |
|  | Cleaned or disinfected living or work spaces more frequently | 1.20 (0.69, 2.09) |
|  | Avoided or decreased public transportation use | 1.36 (0.55, 3.38) |
| Gastrointestinal^d^  (n = 168) | Stayed home | 0.83 (0.56, 1.23) |
|  | Avoided contact with others outside of the household | 1.03 (0.69, 1.54) |
|  | Wore a face mask in public | 0.67 (0.38, 1.18) |
|  | Covered cough or sneeze more frequently | 0.54 (0.36, 0.81) |
|  | Washed or sanitized hands more frequently | 0.87 (0.58, 1.31) |
|  | Cleaned or disinfected living or work spaces more frequently | 1.29 (0.81, 2.04) |
|  | Avoided or decreased public transportation use | 0.82 (0.36, 1.91) |
| Influenza-like illness, ILI^e^  (n = 158) | Stayed home | 1.24 (0.80, 1.94) |
|  | Avoided contact with others outside of the household | 1.01 (0.63, 1.62) |
|  | Wore a face mask in public | 0.60 (0.28, 1.3) |
|  | Covered cough or sneeze more frequently | 1.31 (0.79, 2.18) |
|  | Washed or sanitized hands more frequently | 0.81 (0.51, 1.30) |
|  | Cleaned or disinfected living or work spaces more frequently | 1.29 (0.72, 2.30) |
|  | Avoided or decreased public transportation use | 2.76 (1.29, 5.91) |
| COVID-19-like illness, CLI 1^f^  (n = 107) | Stayed home | 0.73 (0.44, 1.24) |
|  | Avoided contact with others outside of the household | 0.58 (0.33, 1.03) |
|  | Wore a face mask in public | 0.79 (0.30, 2.13) |
|  | Covered cough or sneeze more frequently | 0.73 (0.26, 2.03) |
|  | Washed or sanitized hands more frequently | 0.68 (0.40, 1.18) |
|  | Cleaned or disinfected living or work spaces more frequently | 0.65 (0.32, 1.35) |
|  | Avoided or decreased public transportation use | 1.37 (0.54, 3.44) |
| COVID-19-like illness, CLI 2^g^  (n = 650899) | Stayed home | 0.67 (0.47, 0.95) |
|  | Avoided contact with others outside of the household | 0.79 (0.55, 1.14) |
|  | Wore a face mask in public | 0.83 (0.53, 1.31) |
|  | Covered cough or sneeze more frequently | 1.31 (0.90, 1.89) |
|  | Washed or sanitized hands more frequently | 0.90 (0.63, 1.29) |
|  | Cleaned or disinfected living or work spaces more frequently | 0.81 (0.53, 1.25) |
|  | Avoided or decreased public transportation use | 0.74 (0.38, 1.45) |

^a^ Illness was defined per acute respiratory illness case definition: cough or two qualifying symptoms (fever, sore throat, runny nose, muscle or body aches, headache, difficulty breathing, fatigue, nausea or vomiting; for participants < 18 years of age, ear pain or drainage, rash, and diarrhea were also qualifying symptoms).

^b^ Constitutional syndrome was defined as at least one symptom among fever, fatigue, muscle/body aches, chills, sweats, and headache.

^c^ Respiratory syndrome was defined as at least one symptom among runny nose, sore throat, cough, and trouble breathing.

^d^ Gastrointestinal syndrome was defined as illnesses including nausea/vomiting and/or diarrhea.

^e^ Influenza-like illness was defined per CDC case definition: fever AND cough and/or sore throat.

^f^ COVID-like illness 1 was defined per initial CDC case definition: fever AND symptoms of lower respiratory illness (e.g., cough, shortness of breath).

^g^ COVID-like illness 2 was defined by the following CDC case definition (April 5, 2020): at least two of the following symptoms (fever, chills, rigors, myalgia, headache, sore throat, new olfactory and taste disorder(s)) or at least one of the following symptoms (cough, shortness of breath, or difficulty breathing).
